# Pleural Empyema due to Group D Salmonella

**DOI:** 10.1155/2012/524561

**Published:** 2012-09-29

**Authors:** Jennifer C. Kam, Sami Abdul-Jawad, Chintan Modi, Yazan Abdeen, Fady Asslo, Vikram Doraiswamy, Joseph R. DePasquale, Robert S. Spira, Walid Baddoura, Richard A. Miller

**Affiliations:** ^1^Department of Internal Medicine, St. Michael's Medical Center, School of Graduate Medical Education, Seton Hall University, South Orange, NJ, USA; ^2^Division of Pulmonary Medicine, St. Michael's Medical Center, Seton Hall University, Newark, NJ, USA; ^3^Department of Gastroenterology, St. Michael's Medical Center, Seton Hall University, Newark, NJ, USA; ^4^Division of Gastroenterology, St. Joseph's Regional Medical Center, Seton Hall University, Paterson, NJ, USA

## Abstract

Non-typhi *Salmonella* normally presents as a bacteremia, enterocolitis, and endovascular infection but rarely manifests as pleuropulmonary disease. We present a case of a 66-year-old female with underlying pulmonary pathology, secondary to an extensive smoking history, who presented with a left-sided pleural effusion. The causative agent was identified as being group D *Salmonella*. Decortication of the lung was performed and the patient was discharged on antibiotics with resolution of her symptoms. This case helps to support the inclusion of *Salmonella* group D as a possible etiological agent of infection in the differential causes of exudative pleural effusions.

## 1. Introduction 

 Salmonella is gram-negative, non-spore forming facultative anaerobic motile bacilli. The genus Salmonella belongs to the family of Enterobacteriaceae and is named after Daniel E. Salmon who first isolated *Salmonella cholera suis* from pigs in 1884 [1].   *Salmonella typhi* and *paratyphi* have been the most well-described species as they are known to be the causative agents for typhoid fever. Non-typhi Salmonella species cause a variety of human infections including bacteremia, enterocolitis, and endovascular infections; however, they infrequently cause pleuropulmonary disease. In the absence of concurrent pulmonary infection, a pleural empyema caused by nontyphoid Salmonella is a rare event, with only 16 cases reported to date. We present a case of a unilateral pleural empyema caused by group D Salmonella along with a brief discussion and review of literature of non-typhi Salmonella empyemas.

## 2. Case Presentation

A 66-year-old Hispanic female was admitted with a 3-day history of pleuritic chest pain below the left breast associated with dyspnea on exertion. She reported subjective weight loss but denied any fever, cough, or recent gastrointestinal symptoms. Her past medical history was significant for hypertension, diabetes mellitus (type II), coronary artery disease, and a 20-pack-year smoking history. Physical examination revealed stable vital signs and no jugular venous distension. Respiratory examination was remarkable for dullness to percussion with decreased breath sounds over the left lung base, but no rales or rhonchi were appreciated. Lower extremity edema was noted but no clubbing was present. The remainder of the physical examination was unremarkable.

**Figure 1:**
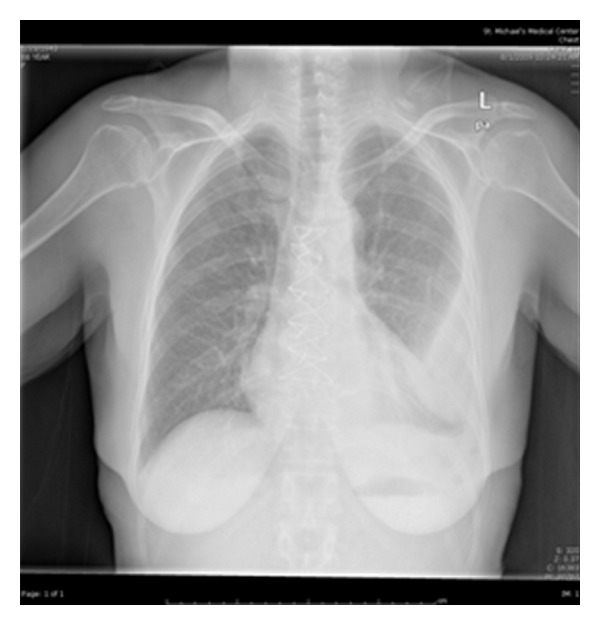
Posteroanterior chest X-ray view of a patient showing opacification of the lower two-thirds of the left hemithorax.

**Figure 2:**
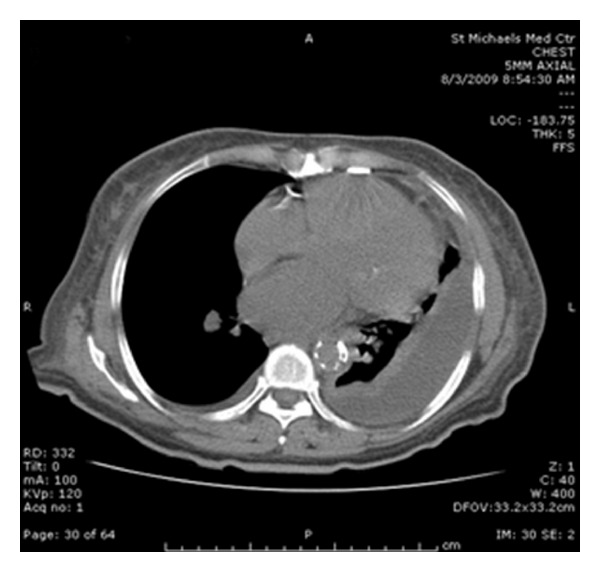
CT scan of the chest showing a large left pleural effusion with loculation.

Initial laboratory findings were significant for a white blood cell count of 11,800/mm^3^ with 84% neutrophils and 9% lymphocytes, BUN 41 mg/dL, and creatinine 2.00 mg/dL. X-ray imaging of the chest showed opacification of the lower two-thirds of the left hemithorax (Figure 1). Computed tomography (CT) scanning of the chest showed a large left pleural effusion that was partially loculated (Figure 2). A thoracentesis was subsequently performed and analysis of the pleural fluid showed the following: amylase 12 U/dL, triglyceride 43 mg/dL, cholesterol 67 mg/dL, glucose 92 mg/dL, lactate dehydrogenase 292 U/L, total protein 4.0 g/dL, and white blood cell count 1033/mm^3^, along with gram-negative cocci bacilli. The pleural fluid was consistent with an exudative effusion according to Lights' criteria with a protein ratio greater than 0.5 and a lactate dehydrogenase ratio greater than 0.6. Vancomycin and Cefepime were initiated for broad-spectrum antibiotic coverage and a chest tube was placed to drain the empyema. The following day, cultures of the pleural fluid grew Salmonella group D and Vancomycin was consequently discontinued. Blood cultures were all negative for any bacterial growth. On the fifth day of admission, the patient underwent decortication of the lung due to loculation of the pleural effusion. Four days thereafter the patient's symptoms resolved and she was discharged on oral Ciprofloxacin.

## 3. Discussion

Salmonella infections typically manifest as gastroenteritis, bacteremia, or septicemia [2]. Extraintestinal complications, such as pleuropulmonary infections, secondary to nontyphoid serotypes of Salmonella are extremely rare, with only a few cases reported in the last century. 

In 1977, a case of Salmonella empyema was reported as a complication of a malignant pleural effusion in an immunocompromised patient. In this case, antimicrobial therapy was found to be most effective when given via intrapleural administration rather than parenterally [3]. In 1978, 2 cases of Salmonella empyemas were reported, with the identified organism being *Salmonella newport*. One patient had concomitant sickle cell disease [4] and the other patient was found to have a splenic abscess [5]. In Italy, in 1984, a case of *Salmonella choleraesuis* pleural empyema was reported in a patient with metastatic breast cancer [6]. That same year, a case of an antibiotic-resistant *Salmonella typhimurium *empyema was reported in a patient with underlying alveolar cell carcinoma [7].

In Spain, 11 cases of pleuropulmonary nontyphoid Salmonella infections were described over a 27-year-period [8]. In the 11 cases described, 8 patients had pneumonia, 2 had a lung abscess, and 1 patient had an empyema. Of these patients, 7 patients were severely immunosuppressed, and 7 had previous lung disease. 

In Virgen de La Concha Hospital in Zamora, Spain, 9 cases were reported over a 5-year-period in which focal soft tissue infections (e.g., abdominal wall abscesses) were the most common features, with a concomitant bacteremia detected in only one patient [9]. A similar case of focal infection was reported in Sydney in a patient with non-Hodgkin's lymphoma [10].

In 1997, Wolday and Seyoumreported a case of a patient with HIV who developed a pleural empyema which was later found to be due to *Salmonella enteritidis* [11].

In 2000, two cases of *Salmonella senftenberg* were acquired while in hospital, one in a patient with diabetes and the other in a patient with gallbladder carcinoma [12]. A case of pleural empyema due to *Salmonella mendoza* was reported in Israel in a patient with myelodysplastic syndrome who developed a splenic abscess [13]. 

According to Crumonly 39 cases of non-typhi Salmonella-infected empyemas had been reported up until 2005 [14]. From 2005 to 2010, only one other case has been reported to our knowledge. This case was presented by Takiguchi et al., where a 65-year-old man with tuberculosis pleuritis was found to have a chronic empyema caused by *Salmonella livingstone* [15].

In differentiating Salmonella subtypes, non-typhi Salmonella is acquired from an animal reservoir unlike *S. typhi* and *paratyphi* whose reservoir is the human. Transmission of non-typhi Salmonella mostly occurs through infected animal food products such as eggs and dairy products. Spreading of the organism can occur via multiple routes: aspiration of gastric secretions of patients with non-typhi Salmonella gastrointestinal infection, seeding from dormant non-typhi Salmonella in the reticuloendothelial system, direct extension from nearby infection such as a splenic abscess, and hematogenous spread [15, 16].

The incidence of developing non-typhi Salmonella appears highest among those who are immunocompromised (i.e., HIV, chronic glucocorticoid therapy, and malignancy) and those who have hemoglobinopathies, such as sickle cell disease [4–7, 9–11, 13, 15, 17, 18]. Patients with infections causing reticuloendothelial system blockage, such as malaria or histoplasmosis, are also at an increased risk as the pathogens can remain dormant before seeding to affected tissues [14]. In patients with pleuropulmonary non-typhi Salmonella infection, 40% have been identified as having prior lung or pleural pathology [14], as is the case with the patient we present here. 

Unlike other extraintestinal non-typhi Salmonella infections, pleuropulmonary disease presents with an acute onset of symptoms. Presenting symptoms are similar to those that occur with pneumonia, including pleuritic pain and shortness of breath. If an empyema is present, cultures from the pleural fluid aid in identifying the causative organism [14]. Blood and stool cultures should also be done in suspected individuals [14–17].

Prolonged antibiotic therapy is the treatment of choice for patients with pleuropulmonary non-typhi Salmonella disease. Antibiotic resistance is increasing among non-typhi Salmonella subtypes; therefore, antibiotic therapy should include a third generation cephalosporin or fluoroquinolone [17]. A 2–4-week course of antibiotics is usually recommended; however, prolonged therapy up to 12 weeks may be necessary in resistant or relapsing infection [11, 15]. Drainage of an empyema is required as adjuvant therapy to antibiotics and decortication may be done in selected patients [15–18].

## 4. Conclusion

We present a case of an exudative pleural effusion caused by group D Salmonella. Salmonella D is a nontyphoid serotype of Salmonella and is an unusual cause of pleuropulmonary infections; however, as our case demonstrates, non-typhi Salmonella should be included in the differential as a causative agent for an exudative pleural effusion.
